# Management of biochemical recurrence after radical prostatectomy for prostate cancer: A case report

**DOI:** 10.1097/MD.0000000000016351

**Published:** 2019-07-05

**Authors:** Jiayan Shen, Shoumei Zang, Xiaokai Yu, Feng Zhao, Peng Jiang, Baishu Zhong, Hua Zhou, Senxiang Yan

**Affiliations:** a Department of Radiation Oncology; b Department of Urology; c Department of Radiology, The First Affiliated Hospital, School of Medicine, Zhejiang University, Hangzhou, China.

**Keywords:** biochemical recurrence (BCR), castration-resistant prostate cancer (CRPC), multiple disciplinary team (MDT), salvage radiation therapy (SRT)

## Abstract

**Rational:**

How to manage patients with prostate cancer (PCa) with biochemical recurrence (BCR) following primary curative treatment is a controversial issue. Multiple disciplinary team (MDT) mechanism may propose an appropriate treatment plan for patients and can effectively improve patient prognosis and survival, reduce patient diagnosis and treatment waiting time, and greatly improve patient satisfaction.

**Patient concerns:**

Here, we presented a case of a 77-year-old man with a persistently elevated serum level of prostate-specific antigen (PSA), who had a history of radical prostatectomy (RP) and of 9 years endocrine therapy.

**Diagnoses:**

Castration-resistant prostate cancer and locally recurrent prostate cancer.

**Interventions:**

Androgen-deprivation therapy was first utilized 2 months after RP, due to the consideration of BCR on May 5, 2007. And during the next 9 years, he was treated with different endocrine agents but failed to maintain serum levels of PSA stable. Finally, the MDT suggested patient to perform salvage radiation therapy (SRT). Under MDT mechanism, we avoid secondary surgery, so as to reduce the patients’ mental suffering and cost of patient care.

**Outcomes:**

EPIC26 scale assessment revealed leak-free urine, good urine control, no defecation abnormalities or blood in the stool, no breast tenderness and breast enlargement significantly improved. The patient now has no adjuvant therapy, including endocrine therapy. The patient achieved good prognosis through local RT.

**Lessons:**

Pelvic SRT for patients with locally recurrent PCa may restore the same radical effect as RP. And more importantly, MDT mechanism plays an important role in making the most appropriate decisions for patients.

## 1 Introduction

Currently, more prostate cancer (PCa) patients depend on multidisciplinary care in managing their disease, as treatment has evolved to become increasingly multidisciplinary, using combinations of surgery, radiation, and/or systemic therapy. The conception of multiple disciplinary team (MDT) was first proposed by the United States in 1990s’.^[1]^ Patients and medical workers can choose the most suitable treatment through multidisciplinary cooperation, and to avoid unnecessary treatment, so as to reduce patients’ mental suffering and cost of patient care. In this study, we present a case report of a 77-year-old man who met the definition of biochemical recurrence (BCR), castration-resistant prostate cancer (CRPC) and local recurrence demonstrated by magnetic resonance imaging (MRI).

## 2 Case presentation

**Figure 1:**
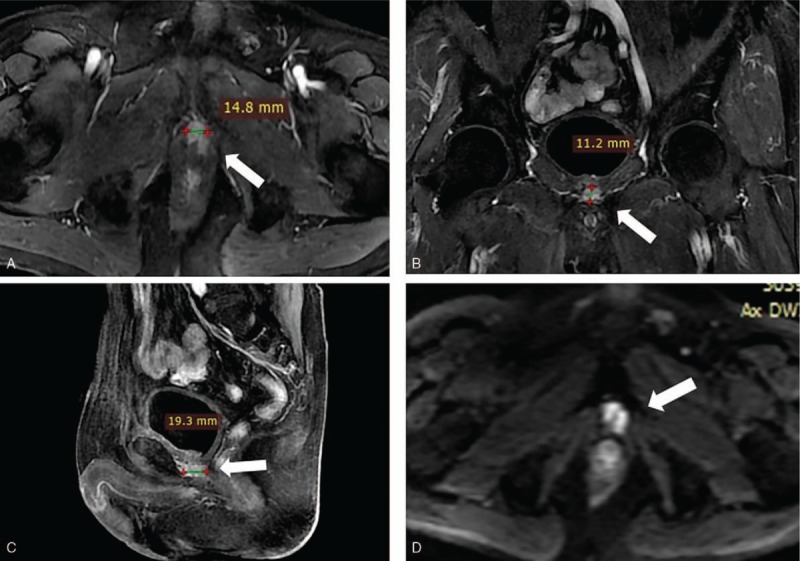
Pelvic MRI (2016-2-22). Follow-up MR images acquired 9 years after initial RP, T2WI MR images (A, B, C) and DW image (D) reveal an irregular nodule (arrow) with high intensity located on resected prostate tumor bed. MRI = magnetic resonance imaging, RP = radical prostatectomy.

A 77-year-old man was seen in the radiation oncology center at our hospital because of a persistently elevated serum level of prostate-specific antigen (PSA) in March 7, 2016. Ten years earlier, he was found to have a PSA level of 52.736 ng/ml at his annual physical examination. And pelvic MRI revealed an evidence of PCa. The reminder of the examination is unremarkable. Thus, a systematic transrectal ultrasound-guided biopsy in February 7, 2007 demonstrated bilateral focus of adenocarcinoma (Gleason score, 9 out of 10 [left side, grade 4 plus grade5]; 7 out of 10 [right side, grade 3 plus grade 4]). The patient was treated with radical prostatectomy (RP) because of the definite diagnosis on March 8, 2007. Surgical pathology showed no tumor invasion in the left and right seminal vesicles and deferens, and no metastasis was found in bilateral iliac lymph nodes, but with bladder neck invasion. So, the pretreatment clinical and postoperative pathologic features suggested that he fell into pT4N0M0 stage and the National Comprehensive Cancer Network high-risk group. Following surgery, the patient was regularly monitored serum level of PSA. And it decreased to 0.001 ng/ml 25 days after RP. However, 2 consecutive follow-up PSA values higher than 0.2 ng/ml approximately 2 months after RP. Casodex was then used as one of an adjuvant therapy at another institution at May 10, 2007, due to consideration of the BCR. During the next 9 years, he was treated with different endocrine agents because of unstable serum levels of PSA, which including leuprorelin, goserelin, flutamide. Nevertheless, the serum level of testosterone was maintained at castration level, but serum levels of PSA still elevated. Until February 22, 2016, the pelvic MRI showed a nodule in the prostate and should be further confirmed whether it was a local recurrence (Fig. 1). After MDT discussion, the current diagnoses of this patient were:

(1) Postoperative PCa, high risk;
(2) CRPC;
(3) local recurrence.

According to the absence of clear guidelines, treatment options at this stage are difficult to choose. Generally, there are several possible treatment options as follows:

(1) continuous androgen-deprivation therapy (ADT);
(2) secondary surgery;
(3) salvage radiation therapy (SRT);
(4) systemic treatment with docetaxel or other medicine, or
(5) other treatments.

**Figure 2:**
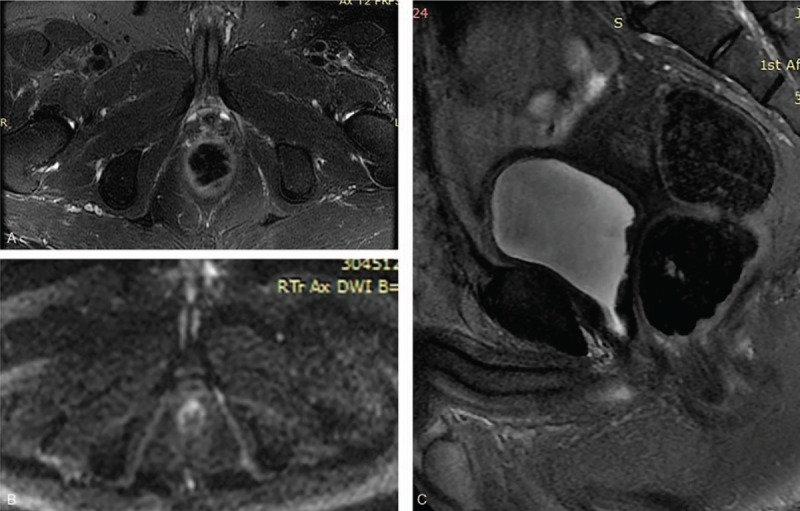
Pelvic MR images (2017-6-26). Follow-up MR images acquired 1 year after SRT, no significant lesion was seen on T2WI MR images (A, C) and DW image (B) on resected prostate tumor bed. SRT = salvage radiation therapy.

**Figure 3:**
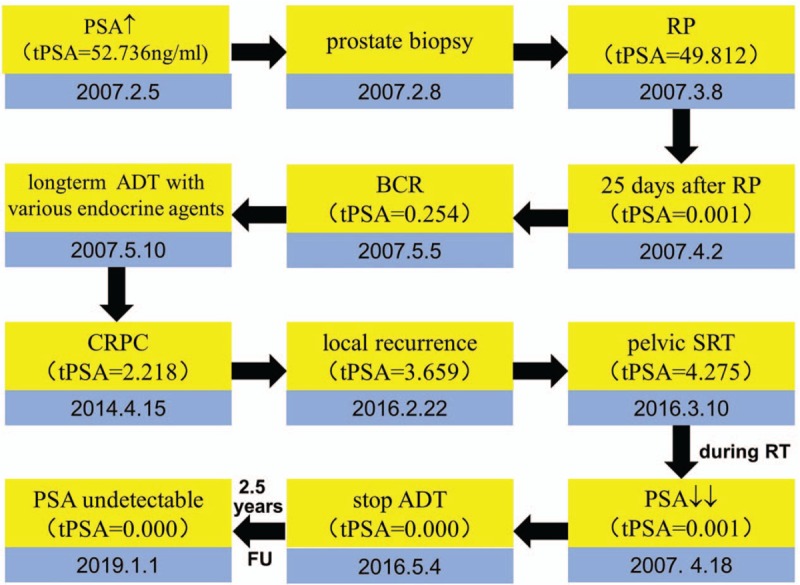
Patient's treatment timeline. 1.5 y = 1.5 years, ADT = androgen-deprivation therapy, BCR = biochemical recurrence, CRPC = castration-resistant prostate cancer, FU = follow up, PSA = prostate-specific antigen, RP = radical prostatectomy, SRT = salvage radiation therapy.

In our institution, PCa MDT was carried out for this patient, and then the SRT was suggested for the patients. Therefore, the patient underwent pelvic RT from March 10, 2016 to May 3, 2016 with a total dose of 200 cGy × 37 F = 7400 cGy. The target area was the prostate tumor bed area + local recurrence area. Each stage of field modification was based on diffusion images of the patient with MR, in order to minimize the doses of organs at risk, while maximizing the radiation doses of locally recurrent lesions. After 37 fractions of RT treatment, he underwent follow-up examinations every 3 months. The serum levels of PSA were all between 0 and 0.007 ng/ml, and serum levels of testosterone were between 41.9 and 188.7 ng/dl. After 1 year of RT, the pelvic MRI scan in June 26, 2017 showed no evidence of nodules in the prostate region and no enlarged lymph nodes were found in the pelvic cavity (Fig. 2). And EPIC26 scale assessment revealed leak-free urine, good urine control, no defecation abnormalities or blood in the stool, no breast tenderness and breast enlargement significantly improved. The patient now has no adjuvant therapy, including endocrine therapy. Quality of life intact, and is still under follow-up, the specific course of disease and related treatment shown in Figure 3.

## 3 Discussion

How to manage patients with PCa with BCR following primary curative treatment is a controversial issue. The options for treatment of recurrence after RP are, according to the EAU, RT at least to the prostatic bed, complete or intermittent ADT, or observation.^[2]^ This patient adopted continuous ADT when he presented to his local urologist. Until he was diagnosed with CRPC and MRI demonstrated local recurrence did he came to our radiation oncology center for advice. Through multidisciplinary discussion, we found that systemic treatment with docetaxel or other medicine for him has little overall survival benefit, but high economic costs.^[3–7]^ So we began to turn to the local treatments. For local treatments, choose secondary surgery or SRT? It is reported that locally recurrent postoperative PCa may have a possible secondary surgical removal of the recurrent focal, but most of the cases of recurrent tumor produced obstruction complications at the same time, such as the involvement of ureteral causing kidney seeper, or involvement of the bladder urethral anastomotic causing dysuria, and so on.^[8]^ However, the patient was 77 years old with a single recurrent focus and no local obstruction, and the patient and his families both refused surgery. Therefore, after evaluating the patient's systemic symptoms and reviewing the relevant literature, we believe that the patient is considered unsuitable for second surgery. Finally, pelvic local SRT was performed for him. For this patient, in the positioning before RT and before each RT, bladder volume was used to measure the urinary volume in the bladder, which was basically controlled at the same level to reduce the internal target volume and further protect the organ at risk. For patients with good prognosis, although RP can achieve better results, but there are still about 40% of patients relapse after surgery, of whom approximately 15% will eventually die of local recurrence or distant metastasis.^[9,10]^ PSA monitoring can prompt the relapse 6 to 48 months in advance, the serum PSA levels more than 2 consecutive ≥0.2 ng/ml defined as BCR. Therefore, patients with PCa after radical surgery may appear PSA gradually increased, BCR, and then enter the CRPC stage, and finally the recurrence of PCa and distant metastasis process.

In conclusion, the patient has been progression-free for 30 months up to now. Therefore, we have reasons to believe that pelvic SRT for patients with locally recurrent PCa can restore the same radical effect as RP. Accordingly, for such postoperatively recurrent PCa, it should be actively carried out early pelvic SRT, thereby blocking the progression of PCa, so that patients regain the effect of cure, and expect to get a better prognosis. And more importantly, under MDT mechanism, it can effectively improve patient prognosis and survival, reduce patient diagnosis and treatment waiting time, and greatly improve patient satisfaction.

## Author contributions

**Conceptualization:** Feng Zhao, Senxiang Yan.

**Data curation:** Jiayan Shen, Baishu Zhong, Hua Zhou.

**Investigation:** Jiayan Shen, Shoumei Zang.

**Methodology:** Feng Zhao, Senxiang Yan.

**Resources:** Jiayan Shen, Xiaokai Yu, Peng Jiang, Baishu Zhong, Hua Zhou.

**Supervision:** Feng Zhao, Senxiang Yan.

**Writing – original draft:** Jiayan Shen, Shoumei Zang, Xiaokai Yu, Peng Jiang.

**Writing – review and editing:** Feng Zhao, Senxiang Yan.

## References

[1] Slavova-Azmanova NS Johnson CE Platell C Peer review of cancer multidisciplinary teams: is it acceptable in Australia? Med J Aust 2015;202:144–7. 25669477 10.5694/mja14.00768

[2] Mottet N Bellmunt J Bolla M EAU-ESTRO-SIOG guidelines on prostate cancer. Part 1: screening, diagnosis, and local treatment with curative intent. Eur Urol 2017;71:618–29. 27568654 10.1016/j.eururo.2016.08.003

[3] Tannock IF de Wit R Berry WR Docetaxel plus prednisone or mitoxantrone plus prednisone for advanced prostate cancer. N Engl J Med 2004;351:1502–12. 15470213 10.1056/NEJMoa040720

[4] de Bono JS Oudard S Ozguroglu M Prednisone plus cabazitaxel or mitoxantrone for metastatic castration-resistant prostate cancer progressing after docetaxel treatment: a randomised open-label trial. Lancet 2010;376:1147–54. 20888992 10.1016/S0140-6736(10)61389-X

[5] Kantoff PW Higano CS Shore ND Sipuleucel-T immunotherapy for castration-resistant prostate cancer. N Engl J Med 2010;363:411–22. 20818862 10.1056/NEJMoa1001294

[6] Beer TM Armstrong AJ Rathkopf DE Enzalutamide in metastatic prostate cancer before chemotherapy. N Engl J Med 2014;371:424–33. 24881730 10.1056/NEJMoa1405095 PMC4418931

[7] Ryan CJ Smith MR Fizazi K Abiraterone acetate plus prednisone versus placebo plus prednisone in chemotherapy-naive men with metastatic castration-resistant prostate cancer (COU-AA-302): final overall survival analysis of a randomised, double-blind, placebo-controlled phase 3 study. Lancet Oncol 2015;16:152–60. 25601341 10.1016/S1470-2045(14)71205-7

[8] Crain DS Amling CL Kane CJ Palliative transurethral prostate resection for bladder outlet obstruction in patients with locally advanced prostate cancer. J Urol 2004;171:668–71. 14713783 10.1097/01.ju.0000104845.24632.92

[9] Brockman JA Alanee S Vickers AJ Nomogram predicting prostate cancer-specific mortality for men with biochemical recurrence after radical prostatectomy. Eur Urol 2015;67:1160–7. 25301759 10.1016/j.eururo.2014.09.019 PMC4779062

[10] Boorjian SA Thompson RH Tollefson MK Long-term risk of clinical progression after biochemical recurrence following radical prostatectomy: the impact of time from surgery to recurrence. Eur Urol 2011;59:893–9. 21388736 10.1016/j.eururo.2011.02.026

